# Evaluation of COVID-19 vaccines in primary prevention against infections and reduction in severity of illness following the outbreak of SARS-CoV-2 omicron variant in Shanghai

**DOI:** 10.3389/fmed.2023.1079165

**Published:** 2023-02-08

**Authors:** Dawei Yang, Huifen Weng, Rui Wang, You Li, Hao Zhang, Shifeng Shao, Hunan Huang, Yuanlin Song, Xiaoyan Chen, Dongni Hou, Yin Wu, Xingwei Lu, Wei Yang, Zhengguo Chen, Xiaohan Hu, Jianwei Xuan, Chunxue Bai, Yaoli Wang

**Affiliations:** ^1^Department of Pulmonary and Critical Care Medicine, Zhongshan Hospital, Fudan University, Shanghai, China; ^2^Shanghai Engineer and Technology Research Center of Internet of Things for Respiratory Medicine, Shanghai Respiratory Research Institution, Shanghai, China; ^3^Shanghai Suvalue Healthcare Scientific Co., Ltd., Shanghai, China; ^4^State Key Laboratory of Trauma, Burns and Combined Injury, Wound Trauma Medical Center, Institute of Surgery Research, Daping Hospital, Army Medical University, Chongqing, China; ^5^Hospital of the People's Liberation Army Joint Logistics Support Force, Yingtan, Jiangxi, China; ^6^Shanghai Key Laboratory of Lung Inflammation and Injury, Shanghai Institute of Infectious Disease and Biosecurity, Shanghai, China; ^7^School of Pharmaceutical Sciences, Health Economic Research Institute, Sun Yat-sen University, Guangzhou, China; ^8^Shanghai Centennial Scientific Co., Ltd., Shanghai, China; ^9^Department of Clinical Research Management Office, The Third Affiliated Hospital of Guangzhou Medical University, Guangzhou, China

**Keywords:** COVID-19, vaccines, epidemiology, public health, respiratory tract infections, health policy

## Abstract

**Objectives:**

To evaluate COVID-19 vaccines in primary prevention against infections and lessen the severity of illness following the most recent outbreak of the SARS-CoV-2 Omicron variant in Shanghai.

**Data sources:**

Data from 153,544 COVID-19 patients admitted to the Shanghai “Four-Leaf Clover” Fangcang makeshift shelter hospital were collected using a structured electronic questionnaire, which was then merged with electronic medical records of the hospital. For healthy controls, data on vaccination status and other information were obtained from 228 community-based residents, using the same structured electronic questionnaire.

**Methods:**

To investigate whether inactivated vaccines were effective in protecting against SARS-CoV-2 virus, we estimated the odds ratio (OR) of the vaccination by comparing cases and matched community-based healthy controls. To evaluate the potential benefits of vaccination in lowering the risk of symptomatic infection (vs. asymptomatic), we estimated the relative risk (RR) of symptomatic infections among diagnosed patients. We also applied multivariate stepwise logistic regression analyses to measure the risk of disease severity (symptomatic vs. asymptomatic and moderate/severe vs. mild) in the COVID-19 patient cohort with vaccination status as an independent variable while controlling for potential confounding factors.

**Results:**

Of the 153,544 COVID-19 patients included in the analysis, the mean age was 41.59 years and 90,830 were males (59.2%). Of the study cohort, 118,124 patients had been vaccinated (76.9%) and 143,225 were asymptomatic patients (93.3%). Of the 10,319 symptomatic patients, 10,031 (97.2%), 281 (2.7%), and 7 (0.1%) experienced mild, moderate, and severe infections, respectively. Hypertension (8.7%) and diabetes (3.0%) accounted for the majority of comorbidities. There is no evidence that the vaccination helped protect from infections (OR = 0.82, *p* = 0.613). Vaccination, however, offered a small but significant protection against symptomatic infections (RR = 0.92, *p* < 0.001) and halved the risk of moderate/severe infections (OR = 0.48, 95% CI: 0.37–0.61). Older age (≥60 years) and malignant tumors were significantly associated with moderate/severe infections.

**Conclusion:**

Inactivated COVID-19 vaccines helped provide small but significant protection against symptomatic infections and halved the risk of moderate/severe illness among symptomatic patients. The vaccination was not effective in blocking the SARS-CoV-2 Omicron Variant community spread.

## Introduction

Since the second half of 2020, Shanghai has experienced several low-level community-transmitted COVID-19 outbreaks. However, the city’s daily operations have not been greatly affected due to targeted epidemic-control measures. In the first half of 2022, a major COVID-19 outbreak occurred, driven by the severe acute respiratory syndrome coronavirus 2 (SARS-CoV-2) Omicron variant, spreading quickly at the community level. The sentinel community-transmitted case of unknown origin was reported on 1st March by the Shanghai Municipal Health Commission (1). This was followed by at least two more transmission routes occurring almost simultaneously, beginning at Yonghui Supermarket in Songjiang District, and Huating Hotel (2, 3). A majority of new cases were traced *via* these three epidemiological chains, suggesting that this new SARS-CoV-2 variant was highly contagious and outmaneuvered the previously effective epidemic-control measures. To contain the outbreak more rapidly, and to realize the goal of zero community transmission, Shanghai imposed city-wide lockdown from 28th March onwards.

B.1.1.529, a new variant of SARS-CoV-2, was first detected from specimens collected on 14th November 2021, in South Africa. In 2 weeks, this variant spread rapidly and became the dominant strain of SARS-CoV-2 in South Africa (4). On 26th November, the WHO designated B.1.1.529 as the fifth variant of concern (VOC), or Omicron (5). Currently, at least five major Omicron sub-variants have been reported: BA.1, BA.2, BA.3, BA.4, and BA.5 (6). Among them, BA.1 is currently the globally dominant strain, with BA.2 expected to eventually replace it in an increasing number of countries, while the other sub-variants account for a smaller proportion (7). Statistics showed that the basic reproductive number (R0) of the SARS-CoV-2 Delta variant ranged from 3.2 to 8 (8), while the transmissibility of the Omicron BA.1 sub-variant was about 3.2 times greater than that of Delta, with a doubling time of 3 days (9, 10).

Omicron BA.2, the dominant variant in the outbreak in Shanghai, has high transmissibility, which is 1.4 times greater than that of BA.1 (11, 12). Statistics showed that the household infection rate was higher for BA.2 than BA.1, at 13.4 and 10.3%, respectively (13). Despite the enhanced transmissibility, the Omicron strain has decreased virulence. Studies in many countries found that the symptoms developed in patients infected with Omicron were not typical (14, 15), and the risks of hospitalization, severe illness, or death were all lower than that of earlier dominant variants (4, 16–18). Basic research suggested that Omicron replicated faster in the upper respiratory system but less efficiently in the lungs (19, 20). This partly explains the reduced severity of pulmonary damage caused by Omicron, including fewer cases with severe pneumonia and dyspnea, as well as lower case fatality rates. In addition, the Omicron variant features characteristics that increase immune escape. In other words, it is capable of evading some of the immunity provided by vaccines or prior infections with the other SARS-CoV-2 variants (21–25). Studies concluded that Omicron BA.2 and BA.1 sub-variants had similar immune-escape abilities (13, 26). In a recent small sample study, BA.2 was also found to infect patients who had recovered from a previous infection with BA.1 (26). It is the atypical clinical manifestations and the strong immune-escape ability that led to the stealthy and fast spread of the Omicron strain.

Policies for COVID-19 epidemic control varied significantly by the country during the COVID-19 pandemic, mainly concerning epidemic-control approaches, testing, and vaccination. Unlike many other countries, China has adopted a dynamic zero-case policy (27), where comprehensive efforts are conducted to carry out epidemic monitoring, contact tracing, screening, and quarantine of the infected in designated makeshift shelter medical facilities to curb the spread of the virus. During this outbreak in Shanghai, multiple rounds of mass PCR testing in conjunction with antigen self-tests were implemented to ensure that no cases were left unidentified. At present, China has granted conditional approval for the inactivated COVID-19 vaccines (28), all patients in the study received only the inactivated vaccine, conditionally approved for marketing manufactured by Sinopharmed China Biologics Beijing Institute of Biological Products Co., LTD. (Beijing Institute), Wuhan Institute of Biological Products Co., LTD. (Wuhan Institute) and Beijing Kexing Zhongwei Biotechnology Co., LTD. (Kexing Zhongwei)). As of 24th June 2022, more than 1.1 billion people (around 80%) in China have been vaccinated against COVID-19 (29), a rate which was higher than the global vaccination rate (66.4%) (30). Therefore, the public health context of the population we studied differed from those of previous research.

Based on real-world, large-scale population data, we reviewed the clinical and demographic profiles of COVID-19 patients and healthy controls in Shanghai to investigate whether the vaccination protected people against SARS-CoV-2 infections and its impact on the severity level and disease outcomes.

## Methods

### Database creation

Since the start of the latest outbreak of SARS-CoV-2 in Shanghai, we have systematically collected data from patients infected with SARS-CoV-2 virus and admitted to the ‘Four-leaf Clover’ Fangcang makeshift shelter hospital (which was converted from the National Exhibition and Convention Centre, NECC; referred to as NECC Fangcang hospital hereafter) using a structured electronic questionnaire. Registered data included demographic and clinical characteristics, results of SARS-CoV-2 testing and COVID-19 vaccination, etc. The database was then linked to the hospital’s electronic medical records (EMRs) for extraction of additional information routinely recorded during the stay at a hospital. The data on demographic and clinical characteristics, results of SARS-CoV-2 testing, and COVID-19 vaccination were obtained from community-based healthy controls, using the same structured electronic questionnaire described above.

### Study population and design

The study population is composed of (1) all diagnosed COVID-19 patients confirmed by PCR testing: one positive testing of a single tube and admitted to NECC Fangcang hospital between 9th April and 30th May 2022, and (2) 228 community-based healthy controls from a sample of (>18 years of age) community-based residents who were tested negative in two consecutive samples, separated by at least 24 h. Of the 280 eligible controls selected, 52 declined to participate. Each control was then matched to two COVID-19 cases. Those who received at least one shot of approved vaccines were considered vaccinated. For symptomatic infections, the severity of illness was grouped into three categories: mild, moderate, and severe as defined by the Protocol for Diagnosis and Treatment of COVID-19 (Trial 9th edition) (Appendix Table 1A). Patients or the public were not involved in the design, conduct, reporting, or dissemination plans of our research.

To analyze whether inactivated vaccines were effective in protecting against infection by the SARS-CoV-2 virus, we estimated the odds ratio (OR) of the vaccination by comparing community-based healthy controls with matched cases. To examine the potential benefits of vaccines in preventing symptomatic infection (vs. asymptomatic) and lessening moderate/severe illness (vs. mild), we estimated relative risk (RR) and OR as appropriate among patients infected with SARS-CoV-2 and patients with symptomatic infections between the vaccinated and unvaccinated.

The study protocol was approved by the Ethics Committee of Zhongshan Hospital Fudan University [IRB No. B2022-183(2)] and was registered with the Chinese Clinical Trials Registry and written informed consent was obtained for all study participants electronically.

### Statistical analysis

In the case–control study, selected COVID-19 cases were matched to each healthy control in the ratio of 2:1 using propensity score matching (PSM). The individual propensity score was generated from a logistic regression model using infection status as the dependent variable and age, gender, marital status, hypertension, and diabetes as explainable variables. The cases were matched to controls using the nearest method, i.e., for each control, two cases of nearest neighbors were selected based on their propensity scores.

Enumeration data were described as percentages, and measurement data were described as means with their standard deviation. In single-factor analysis, we estimated the OR of the vaccination status between the case and control groups and the RR of symptomatic infections between the vaccinated and unvaccinated. To control for potential confounding factors, we used a stepwise logistic regression analysis to determine the OR of symptomatic infections (vs. asymptomatic) with independent variables including vaccination, gender, age, marital status, and co-morbid conditions such as hypertension, diabetes, coronary artery disease, etc. To investigate the potential benefits of the vaccination in lessening moderate/severe infection (vs. mild) in patients with symptomatic infections with controlling for potential confounding factors, we estimated adjusted ORs by applying the stepwise logistic regression analysis with the same independent variables described above. Both models are fitted by the backward direction stepwise approach with all potential confounding factors being included in the initial step.

As our study is descriptive with no prior hypothesis for testing, we did not estimate the required sample size. We conducted the data processing and analysis using RStudio version 3.5.3.

## Results

### Demographics and baseline characteristics

**Figure 1:**
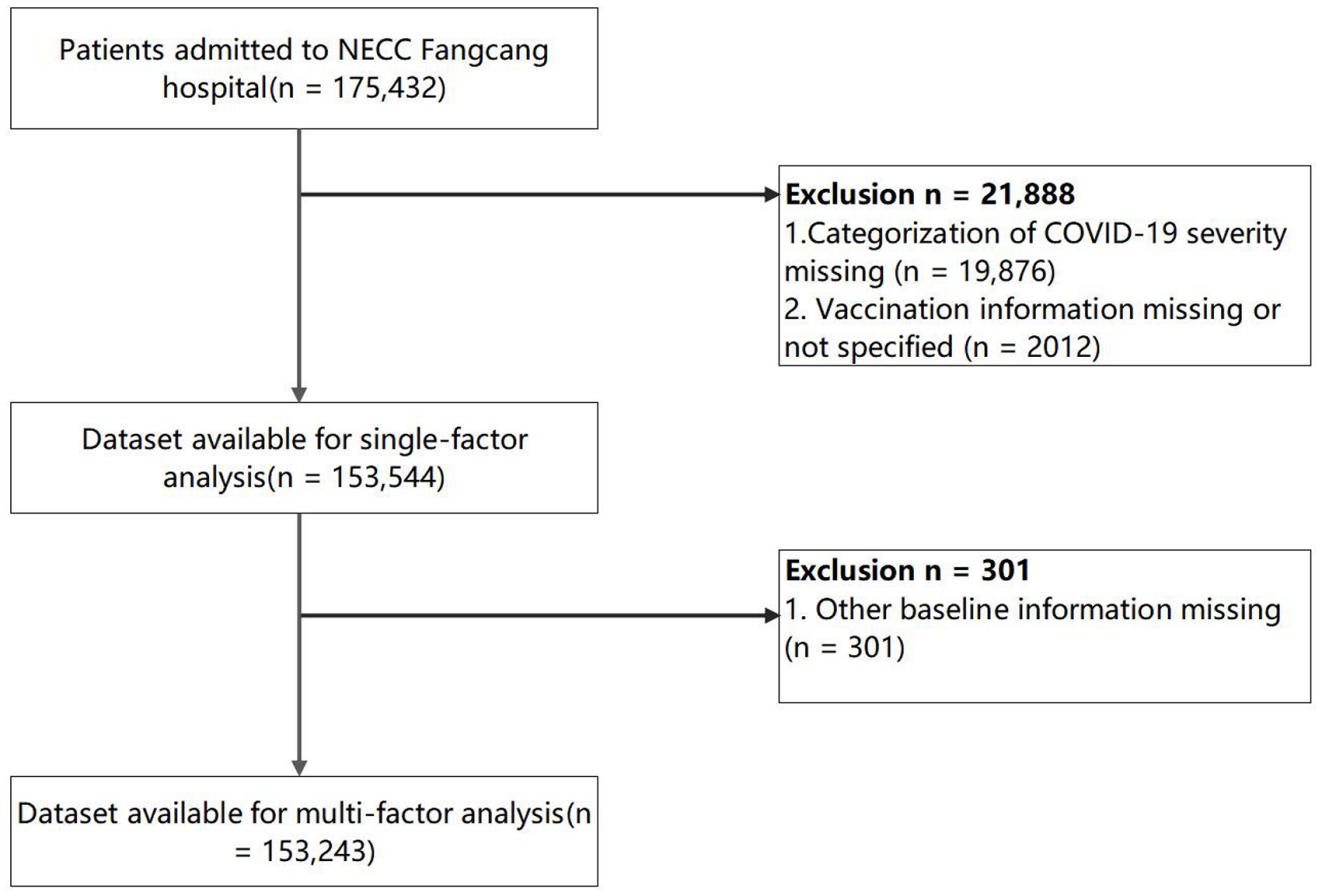
Flow chart of NECC Fangcang hospital data cleaning.

The initial search identified 175,432 patients, after the database construction and cleaning, a total of 153,544 COVID-19 patients were included in the data analysis with the exclusion of the categorization of COVID-19 severity missing (*n* = 19,876) and the vaccination information missing or not specified (*n* = 2,012). The cleaning of the data from the NECC Fangcang hospital is illustrated in the flow chart (Figure 1). In addition, we recruited 228 community-based healthy controls with 456 cases matched from the COVID-19 patient cohort following the PSM. After matching, the cases and controls had nearly identical demographic profiles (mean age: 36.8 vs. 36.8; female gender: 43.9% vs. 44.7%; married: 64.9% vs. 66.2%).

### Single-factor analysis

The baseline characteristics of the COVID-19 patient cohort were included in the analysis (Table 1). Of the 153,544 COVID-19 patients, the mean age was 41.59 years (SD = 15.53) and 90,830 (59.2%) were males. Of the entire patient cohort, 118,124 (76.9%) had been vaccinated and among them, 5,088(3.3%), 47,532 (31.0%%), and 65,504 (42.7%) received one dose, two doses, and three doses (three doses mean the 1st booster after completion of the primary series of 2 vaccinations), respectively. A vast majority of infected persons were asymptomatic (93.3%). Of the 10,319 symptomatic patients, 10,031(97.2%), 281(2.7%), and 7(0.1%) experienced mild, moderate, and severe infections, respectively. Of the 288 moderate/severe symptomatic patients, 158(54.9%) had been vaccinated, which was at a lower rate than those vaccinated in the entire patient cohort (76.9%). Hypertension (8.7%) and Diabetes (3.0%) accounted for the majority of comorbidities. Furthermore, among the 21,084 elder people (age > 60 years), the distribution of age 60–69, 70–79, and ≥80 was 17,157(83.0%), 3,280 (15.9%), 229 (1.1%), respectively. There were also statistically significant (*p* < 0.05) differences in the accumulation of symptoms among patients age 60–69 under different vaccination statuses. However, the difference in the distribution of clinical symptoms between vaccination and non-vaccination could not be considered statistically significant among the 70–79 and ≥80 groups (*p* > 0.05) (Appendix Table 8A).

**Table 1:** Baseline characteristics.

Characteristics	Overall (*n* = 153,544)
Sex, *n* (%)	
Male	90,830 (59.2)
Female	62,714 (40.8)
Age, mean (*SD*)	41.59 (15.53)
Age ≥ 60, *n* (%)	20,666 (13.5)
60–69	17,157 (83.0)
70–79	3,280 (15.9)
≥80	229 (1.1)
Marital status, *n* (%)	
Married	91,919 (59.9)
Unmarried	56,029 (36.5)
Others^#^	5,595 (3.6)
Days of admission, mean (*SD*)	6.68 (3.23)
Vaccination, *n* (%)	118,124 (76.9)
Doses administered, *n* (%)	
0	35,420 (23.1)
1	5,088 (3.3)
2	47,532 (31.0)
3*	65,504 (42.7)
Underlying conditions, *n* (%)	
Hypertension	13,411 (8.7)
Diabetes	4,557 (3.0)
Allergy	3,356 (2.2)
Coronary artery disease	2,770 (1.8)
Stroke	898 (0.6)
Arrhythmia	2,390 (1.6)
Heart failure	822 (0.5)
Peripheral vascular disease	1,390 (0.9)
Malignant tumors	274 (0.2)
Post-operation	365 (0.2)
Renal disease	228 (0.1)
Categorization upon admission, *n* (%)	
Asymptomatic	143,225 (93.3)
Mild	10,031 (6.5)
Moderate	281 (0.2)
Severe	7 (0.0)
Moderate/Severe	
Vaccinated, *n* (%)	158 (54.9)
Unvaccinated, *n* (%)	130 (45.1)

^#^Including divorced, widowhood and people who do not remark the marital status. *3 dose means the 1st booster after completion of the primary series of 2 vaccinations.

We also obtain the distribution of vaccination status of the cases and controls. There was no evidence indicating that the vaccination provided primary prevention against infections (Table 2). Of the 456 cases, 427 (93.6%) had a history of the vaccination and among 228 controls, 216 (94.7%) had received COVID-19 vaccines (OR = 0.82, *p* = 0.613).

**Table 2:** Distribution of vaccination status for both cases and controls.

Vaccination status	Case group (infected patients)	Control group (healthy community residents)	Total	OR	*p*-value
Vaccinated	427	216	643		
Unvaccinated	29	12	41	0.82	0.613
Total	456	228	684		

To evaluate the efficacy of vaccination in patients infected with SARS-CoV-2, we used the symptom status of patients upon admission grouped by vaccination status. As is shown in Table 3, the inactivated vaccines appeared to offer a small but significant protection against symptomatic infections. Among 118,124 vaccinated patients, 7,787 (6.6%) had COVID-19-related symptoms compared to 2,532 (7.1%) patients with symptomatic infections among 35,420 unvaccinated patients (RR = 0.92, *p* < 0.001). Furthermore, the same results were found in the 60–69 years group (RR = 0.85, *p* < 0.001). However, there was no evidence to show that the inactivated vaccines would reduce or increase the relative risk of symptomatic infections among patients aged 70–79 or ≥80 years (RR = 0.98, RR = 1.02, respectively, both *p* > 0.05) (Appendix Table 7A).

**Table 3:** Occurrence of any COVID-19 related clinical symptoms by vaccination status.

	Vaccinated	Unvaccinated	Total	RR	*P*-value
Symptomatic	7,787	2,532	10,319		
Asymptomatic	110,337	32,888	14,3,225	0.92	<0.001
Total	118,124	35,420	153,544		

### Multivariate analysis

A multivariate stepwise logistic regression analysis was applied to evaluate the significant independent predictors for symptomatic SARS-CoV-2 infections upon controlling potential confounding factors. The results showed that receiving inactivated vaccines helped reduce symptomatic infections by 7% (OR = 0.93, 95% CI: 0.88–0.97) (Table 4). One dose, 2 doses of vaccination, and the presence of coronary artery disease were also associated with a lower risk of symptomatic infections (OR = 0.83, 95% CI:0.73–0.94; OR = 0.90, 95% CI:0.85–0.95; OR = 0.82, 95% CI: 0.69–0.97, respectively; Appendix Table 4A and Table 4). Female gender, older age (≥60 years), and post-operation were significantly associated with increased risk of symptomatic infections (OR = 1.23, 95% CI: 1.18–1.28; OR = 1.13, 95% CI: 1.06–1.20; OR = 2.31, 95% CI: 1.70–3.15; respectively; Table 4).

**Table 4:** Stepwise logistic regression analysis of the factors that influenced the presence of symptoms after the patients were infected with SARS-CoV-2.

Influencing factors	OR (95%CI)	*P*-value
Vaccination	0.93 (0.88, 0.97)	0.002
Female	1.23 (1.18, 1.28)	<0.001
Age ≥ 60 years	1.13 (1.06, 1.20)	<0.001
Unmarried	1.03 (0.92, 1.15)	0.606
Married	1.10 (0.99, 1.23)	0.082
Diabetes	0.92 (0.81, 1.03)	0.160
Coronary artery disease	0.82 (0.69, 0.97)	0.022
Peripheral vascular disease	0.83 (0.65, 1.06)	0.140
Post-operation	2.31 (1.70, 3.15)	<0.001
Renal disease	1.54 (0.99, 2.39)	0.055

Finally, to further assess whether the factors associated with moderate/severe infections (vs. mild) in patients with symptomatic infections, another stepwise logistic regression analysis was performed. After adjusting for potential confounding factors, inactivated COVID-19 vaccines significantly reduced moderate/severe infections by about half (OR = 0.48, 95% CI: 0.37–0.61). Female gender was also associated with a lower risk of moderate/severe illness (OR = 0.77, 95% CI: 0.60–0.97) (Table 5). Furthermore, two doses and three doses are also significantly associated with a lower risk of moderate/severe infections (OR = 0.51, 95% CI:0.38, 0.7; OR = 0.45, 95% CI:0.34–0.59, respectively; Appendix Table 5A). On the other hand, older age (≥60 years) and malignant tumors were significantly associated with a higher risk of moderate/severe infections (OR = 4.15, 95% CI: 1.50–11.47; OR = 3.14, 95% CI: 2.43–4.04, respectively; Table 5). Furthermore, people aged over 60 vaccinated or unvaccinated were both significantly associated with a higher risk of moderate/severe illness (OR = 3.5, 95% CI: 2.43–5.12; OR = 2.17, 95%CI = 1.85–3.97). More seriously, unvaccinated people over 60 are 1.3 times more likely to suffer moderate/severe disease than vaccinated people (Appendix Table 6A).

**Table 5:** Stepwise logistic regression analysis of the factors associated with moderate/severe illness in patients with symptomatic infections.

Influencing factors	OR (95%CI)	*P*-value
Vaccination	0.48 (0.37, 0.61)	<0.001
Female	0.77 (0.60, 0.97)	0.030
Age ≥ 60 years	3.14 (2.43, 4.04)	<0.001
Coronary artery disease	1.68 (0.93, 3.05)	0.087
Malignant tumors	4.15 (1.50, 11.47)	0.006

## Discussion

This study was based on data from 153,544 people who were infected with SARS-CoV-2 specific variants and quarantined at one designated shelter hospital during the major outbreak of the SARS-CoV-2 Omicron variant in Shanghai between 9th April and 30th May 2022. We also constructed a case–control sub study by comparing the vaccination status of COVID-19 patients with community-based healthy controls. We found no evidence that the vaccination provided primary prevention against SARS-CoV-2 virus. However, inoculation with inactivated vaccines helped provide small but significant protection against symptomatic infections. We also observed female gender, older age (≥60 years), and patients with post-operation were significant risk factors for symptomatic infections. In addition, the vaccination halved the risk of moderate/severe infection in patients presented with COVID-19-related clinical symptoms. Other significant risk factors for moderate/severe illness included older age (≥60 years) and patients with malignant tumors. There were no deaths observed in the patient cohort in our study, which did not correspond to the reported 0.096% mortality rate by the Health Commission of Shanghai, China. This was mainly because of an admission triage protocol implemented, patients would be transferred to advanced care facilities or the designated hospital *via* an emergency transfer system if their medical conditions became unstable and judged to be life-threatening by their attending physicians.

### Vaccination

In our study population, inactivated vaccines were administered as they were approved by a Chinese regulatory agency. The most cited reasons for not being vaccinated were concerns about vaccination side effects, especially among patients presenting with comorbidities. Other well-documented reasons include that people were informed to only complete vaccination 6 months post chemotherapy and the existence of disinformation about the effectiveness of vaccines and disease. It will be critical that the most credible influencers complement and reinforce the messages shared by the government or the health care policy. Among the vaccinated, over 95% received two and more doses. The results showed that the vaccination had small but significant protection against symptomatic infections, which was similar to the findings of previous research (31). Our study also found that the vaccination effectively halved the risk of moderate/severe illness in patients with symptomatic infections. In Appendix Table 4A, while it appears that one dose of vaccination may be more effective in preventing symptomatic infection compared with 2 or 3 doses, the differences were likely due to random variation reflecting the small sample size, more so for patients receiving only one dose. In fact, the confidence intervals for ORs with 1, 2, 3 doses were overlapping, indicating the estimates of ORs by doses were not significantly different statistically. Other studies reported that after two doses of inactivated vaccines, the risk of hospitalization or death after being infected with SARS-CoV-2 virus increased gradually compared with being recently vaccinated (32), and nearly doubled by 6 months after vaccination (33). Statistical analyses have shown that the relative effectiveness of booster vaccination with BNT162b2 (Pfizer/BioNTech) or mRNA-1,273 (Moderna) following primary vaccination with ChAdOx1-S (AstraZeneca) or BNT162b2 ranged from 85 to 95% (34). Booster shots can substantially reduce the risk of severe illness (35). Another study concluded that after two doses of inactivated whole-virion vaccines, a third heterologous booster of protein subunit vaccine could effectively recall the immunological memory and significantly increase immune responses against the SARS-CoV-2 Delta variant (36). In our study, people who had received the 1st booster after completion of the primary series of 2 vaccinations accounted for over 55% of the vaccinated group, and the protection against moderate/severe illness may indicate the effectiveness of inactivated vaccine booster in lessening severe symptom for patients infected with SARS-CoV-2 virus. Another analysis of Omicron-related epidemiological data from China showed that booster vaccination with inactivated vaccines significantly reduced the rate of severe and critical infections (37). This matches what we found in our study which showed that 1–2 doses of vaccine and the presence of coronary artery disease were associated with a lower risk of symptomatic infections. In addition, 2–3 doses were also associated with a lower risk of moderate/severe infections. However, due to the lack of information on vaccine brands and dates of vaccination, we were unable to further investigate the effectiveness of different brands of inactivated vaccines or the waning effectiveness of those vaccines.

### Risk factors

In this study, older age was an important risk factor associated with both symptomatic infections and moderate/severe infections, which was consistent with many previous findings (38–40). Earlier study results also showed that the risk of COVID-19 death increased by more than 16-fold for people aged over 80 compared with those aged 18–34. In our study, the risk of moderate/severe illness was found to be threefold higher in patients aged over 60 than in those aged less than 60. These results suggest that older populations are highly susceptible to COVID-19 and more active preventive measures plus a more aggressive vaccination strategy should be considered to protect them from serious infections. We found that the male gender was also a significant predictor of moderate/severe illness for patients infected with the SARS-CoV-2 virus, which was consistent with other study results showing that the male gender was associated with hospitalization, ICU admission, mechanical ventilation, and death in COVID-19 patients (35, 36).

Previous studies have shown that severely ill COVID-19 patients often had one or more comorbidities, such as hypertension, diabetes, coronary heart disease, renal disease, malignant tumors, etc. In this study, hypertension and diabetes accounted for most common co-morbid conditions, and only malignant tumors were associated with moderate/severe COVID-19 illness, which was also reported in previous studies (38, 41). The susceptibility of patients with malignant tumors to moderate/severe infections could be explained by the immune abnormalities caused by the tumor itself and/or the immunosuppression caused by cancer chemotherapy as shown in prior research (38). Numerous studies have reported the association of metabolic diseases, including hypertension (41, 42) and diabetes (38, 39, 43, 44), with severe COVID-19 illness. Although the prevalence of these two conditions was relatively high in our study population, the association with COVID-19 severity level was not observed, probably because most of the comorbidities in these patients were well controlled, thus lowering their impact on infections. Prevention and treatment of these chronic, underlying diseases will be an integral measure to reduce the risk of severe illness and to improve the population’s response to the COVID-19 epidemic. Some of the previously reported comorbidities, such as chronic airway diseases (38, 45) (including COPD and bronchiectasis), immunosuppressive conditions (39), or anxiety and fear-related disorders (41), were underrepresented in our study population, making it impossible for us to examine their association with severe COVID-19 illness. Furthermore, owing to the lack of BMI data from EMR records, we were unable to investigate the relationship between obesity and severe infection.

The association of coronary artery disease with poor prognosis in COVID-19 patients remains an unsettled topic. Some studies have shown that coronary artery disease or coronary arteriosclerosis detected by computed tomography were associated with severe illness and death (46–48); others have suggested that this might be related to confounders, such as metabolic diseases and socio-economic factors, and after proper adjustment in the statistical analysis, the association between coronary artery disease and severe illness for patients infected with the SARS-CoV-2 virus, was found to be not significant (49, 50). In this study, we noticed a negative association between coronary heart disease and symptomatic infection, this may because that the COVID-19 patients are prone to abnormal blood clotting and some of these patients with coronary heart disease need anticoagulant secondary prophylaxis and take long-term anticoagulants. And it was also possibly due to some confounders that were not included in the multivariate regression model.

### Strengths

The public health context in which this study was conducted differed from previous ones, mainly in the following aspects. (1) It was a concentrated three-month short-term outbreak in a well-defined population that had been largely vaccinated, without a history of infection or COVID-19 epidemic, which led to a reduction in the confounders that were commonly observed in long-term studies, such as the history of infections, changes in regional epidemics, and changes in vaccination rates. (2) The outbreak was completely driven by the Omicron strain, avoiding the impact of different SARS-CoV-2 variants on the study results. (3) During this outbreak, the strictest public health interventions were carried out, including multiple rounds of mass RT-PCR nucleic acid tests, which help gather most patients, the large resource population of the study, to the Fangcang.

This study was based on real-world data and all patients who tested positive were admitted to the designated facility for observation or treatment. This not only reduced selection bias, non-response bias, and information bias in the evaluation of the risk of the severity level of illness but also provided many candidate risk predictors. The International Classification of Diseases, Tenth Revision, Clinical Modification (ICD-10-CM) system was used for disease diagnosis and coding for co-morbid conditions.

### Limitations

This study also has several limitations, including the following. (1) Since EMR data were used, some risk factors may not have been recorded, for example, BMI, income, and long-term residence in medical and nursing institutions. It is possible that some underlying health conditions were missing or that the underlying diseases of severe patients were recorded more rigorously than those of mild patients. (2) The patients who were infected with SARS-CoV-2 virus might be those with high mobility, fewer co-morbid conditions, and relatively good health, while older populations with more severe underlying diseases accounted for a small proportion in this study due to reduced mobility and lower exposure. (3) Although the Omicron variant is exceedingly contagious, it is unclear if the stringent public health policies enforced also resulted in the “no to little difference” in terms of protection of vaccination against infection seen for cases and controls. (4) The endpoints of this study were the presence of symptoms and moderate/severe infections upon admission, and analysis of death and long-term prognosis were missing. (5) The lack of data regarding types of vaccines and dates of vaccination made it difficult for an in-depth analysis of the effectiveness of vaccination. (6) Our database only included about 1/3 of the total number of COVID-19 patients during this outbreak, thus the study population was only partially representative of the entire infected population in Shanghai.

### Public health and clinical implications

We observed that a vast majority of the COVID-19 patient cohort admitted to the designated quarantine hospital was asymptomatic (93.3%), which was approximately the same as the reported 90% by the Health Commission of Shanghai, China. However, this finding has potentially significant public health implications as it makes epidemiologic tracing more challenging. Subclinical infection is likely to be a major route of community spread for SARS-CoV-2 viral strains and highly frequent population-based screening plus forceful quarantine may be the only viable approaches in blocking the disease transmission. However, this can only be achieved at enormous economic and intangible humanistic cost (stress, anxiety, quality of life, etc.). Although the RT-PCR tests for the diagnosis of COVID-19 has very high fixed technical performance metrics, i.e., sensitivity and specificity (51), implementing PCR testing in a large population with a very low rate COVID infection would yield a relatively high number of false positive cases. While repeating the testing may reduce the false positive rate, it would incur additional financial and humanistic costs. Furthermore, it may lead to an increasingly heavy burden on already strained medical institutions.

We suggest that vaccinated individuals should take more active preventive interventions in response to the COVID-19 pandemic, especially those with high-risk factors for symptomatic infection and severe illness. Furthermore, those effective interventions mainly include reducing exposure by strengthening self-protection and keeping social distance, control of chronic, underlying health conditions, and more active primary and booster vaccination. Active interventions should be taken in populations at high risk and should be given priority in accessing medication before it develops into a severe illness, such as antiviral ones and symptomatic treatment. Awareness of high-risk factors can also help avoid the impact of excessive prevention and control measures on populations at low risk.

## Conclusion

Inactivated COVID-19 vaccines helped provide small but significant protection against symptomatic infections and halved the risk of moderate/severe illness among symptomatic patients. The vaccination was not effective in blocking the community spread of the SARS-CoV-2 virus.

## Data availability statement

The original contributions presented in the study are included in the article/Supplementary material, further inquiries can be directed to the corresponding authors.

## Ethics statement

The studies involving human participants were reviewed and approved by the Ethics Committee of Zhongshan Hospital Fudan University (IRB No. B2022-183(2)). The patients/participants provided their written informed consent to participate in this study.

## Author contributions

DY, HW, XH, and JX were joint first authors. WY and CB obtained funding. CB, XH, JX, and YS designed the study. YiW, YaW, RW, YL, SS, HZ, HH, and ZC collected the data. WY and XL were involved in data cleaning, mortality follow-up, and verification. XL analyzed the data. DY, HW, XC, and DH drafted the manuscript. CB, XH, and JX contributed to the interpretation of the results and critical revision of the manuscript for important intellectual content and approved the final version of the manuscript. All authors have read and approved the final manuscript.

## Funding

This work was supported by Chongqing Science and Technology Bureau Project (CSTC2021 jscx-gksb-N0007), National Natural Science Foundation of China (82170110), Shanghai Pujiang Program (20PJ1402400), Science and Technology Commission of Shanghai Municipality (20DZ2254400 and 20DZ2261200), Shanghai Municipal Science and Technology Major Project (ZD2021CY001), Shanghai Municipal Key Clinical Specialty (shslczdzk02201), and Fujian Province Department of Science and Technology (2022D014).

## Conflict of interest

HW and WY were employed by Shanghai Suvalue Healthcare Scientific Co., Ltd., Shanghai, China. XL was employed by Shanghai Centennial Scientific Co., Ltd., Shanghai, China.

The remaining authors declare that the research was conducted in the absence of any commercial or financial relationships that could be construed as a potential conflict of interest.

## Publisher’s note

All claims expressed in this article are solely those of the authors and do not necessarily represent those of their affiliated organizations, or those of the publisher, the editors and the reviewers. Any product that may be evaluated in this article, or claim that may be made by its manufacturer, is not guaranteed or endorsed by the publisher.
