# Supplementary material for: Evaluation of COVID-19 vaccines in primary prevention against infections and reduction in severity of illness following the outbreak of SARS-CoV-2 omicron variant in Shanghai
Source: Front Med (Lausanne). 2023 Feb 8;10:1079165. doi: 10.3389/fmed.2023.1079165 (PMC9946042; doi:10.3389/fmed.2023.1079165)
Supplement: Supplementary file 1 [file Table_1.pdf]

Appendix

Table 1A COVID-19 Diagnosis and Treatment Protocol (Trial Version 9)

Clinical type	Clinical symptoms
Mild	The clinical symptoms were mild, and no pneumonia was observed on imaging.
Moderate	With the above clinical manifestations, imaging findings of pneumonia.
Severe	Adults meet any of the following criteria :1. Shortness of breath, RR ≥ 30 times/min; 2. In the resting state, oxygen saturation ≤ 93% when inhaling air; Arterial partial pressure of oxygen (PaO2) / oxygen concentration (FiO2) ≤ 300mmHg (1mmHg = 0.133kPa); The PaO2/FiO2 should be calibrated according to the following formula for high altitudes (above 1000m): PaO2/FiO2×[760/ atmospheric pressure (mmHg)]. 4. The clinical symptoms were progressively aggravated, and the lung imaging showed significant lesion progression >50% within 24 ~ 48 hours.

Table 4A Stepwise logistic regression analysis of the factors that influenced the presence of symptoms after the patients were infected with SARS-CoV-2(1dose, 2doses, 3doses)

Influencing factors	OR (95%CI)	P value
**1 dose**	0.83 (0.73, 0.94)	**0.004**
**2 doses**	0.90 (0.85, 0.95)	**<0.001**
3 doses*	0.96 (0.91, 1.01)	0.088
Female	1.23 (1.18, 1.28)	<0.001
Age≥60	1.12 (1.06, 1.19)	<0.001
Unmarried	1.04 (0.93, 1.16)	0.526
Married	1.10 (0.98, 1.22)	0.097
Coronary artery	0.81 (0.68, 0.96)	0.015
Peripheral vascular disease	0.82 (0.64, 1.05)	0.109
Infectious disease	1.85 (1.19, 2.88)	0.006
Post-operation	2.22 (1.62, 3.04)	<0.001

*3 dose means the 1st booster after completion of the primary series of 2 vaccinations

Table 5A: Stepwise logistic regression analysis of the factors associated with moderate/severe illness in patients with symptomatic infections((1dose, 2doses, 3doses)

Influencing factors	OR (95%CI)	P value
1 dose	0.5 (0.22, 1.16)	0.106
2 doses	0.51 (0.38, 0.7)	<0.001
3 doses*	0.45 (0.34, 0.59)	<0.001
Female	0.77 (0.6, 0.97)	0.03
Age≥60 years	3.13 (2.43, 4.04)	<0.001
Coronary artery	1.7 (0.94, 3.08)	0.082
Tumor	4.15 (1.5, 11.46)	0.006

*3 dose means the 1st booster after completion of the primary series of 2 vaccinations

Table 6A: Logistic regression analysis of age≥60 associated with moderate/severe illness in patients Unvaccinated VS. Vaccinated

Influencing factors	OR (95%CI)	P value
Unvaccinated	3.53 (2.43, 5.12)	**<0.001**
Vaccinated	2.71 (1.85, 3.97)	**<0.001**

Table 7A Occurrence of any COVID-19 related clinical symptoms by vaccination status in elder patients

	60-69					70-79					≥80				
	Vaccinated	Unvaccinated	Total	RR	P Value	Vaccinated	Unvaccinated	Total	RR	P Value	Vaccinated	Unvaccinated	Total	RR	P Value
Symptomatic	799	547	1,346			108	92	200			3	10	13		
Asymptomatic	10030	5781	15,811	0. 85	<0. 001	1682	1398	3,080	0. 98	>0. 05	49	167	216	1. 02	>0. 05
Total	10,829	6,328	17,157			1,790	1,490	3,280			52	177	229		

Table 8A COVID-19 related clinical symptoms by vaccination status
In the elder patients based on four categories (Asymptomatic, Mild, Moderate, Severe)

	60-69				70-79				≥80			
	Vaccinated	Unvaccinated	Total	P Value	Vaccinated	Unvaccinated	Total	P Value	Vaccinated	Unvaccinated	Total	P Value
Asymptomatic	10030	5781	15811		1682	1398	3080		49	167	216	
Mild	751	495	1246		103	85	188		3	10	13	
Moderate	47	52	99	<0. 05	4	7	11	>0. 05	0	0	0	>0. 05
Severe	1	0	1		1	0	1		0	0	0	
Total	10829	6328	17157		1790	1490	3280		52	177	229	
